# Designing and implementing a socioeconomic intervention to enhance TB control: operational evidence from the CRESIPT project in Peru

**DOI:** 10.1186/s12889-015-2128-0

**Published:** 2015-08-21

**Authors:** Tom Wingfield, Delia Boccia, Marco A. Tovar, Doug Huff, Rosario Montoya, James J. Lewis, Robert H. Gilman, Carlton A. Evans

**Affiliations:** Innovación Por la Salud Y Desarrollo (IPSYD), Asociación Benéfica PRISMA, Lima, Peru; Innovation For Health And Development (IFHAD), Infectious Diseases & Immunity, Imperial College London, and Wellcome Trust Imperial College Centre for Global Health Research, London, UK; The Monsall Infectious Diseases Unit, North Manchester General Hospital, Manchester, UK; Department of Infectious Disease Epidemiology, London School of Hygiene & Tropical Medicine, London, UK; Innovation For Health And Development (IFHAD), Laboratory of Research and Development, Universidad Peruana Cayetano Heredia, Lima, Peru; Tulane University School of Public Health and Tropical Medicine, New Orleans, LA USA; Johns Hopkins Bloomberg School of Public Health, Baltimore, MD USA

## Abstract

**Background:**

Cash transfers are key interventions in the World Health Organisation’s post-2015 global TB policy. However, evidence guiding TB-specific cash transfer implementation is limited. We designed, implemented and refined a novel TB-specific socioeconomic intervention that included cash transfers, which aimed to support TB prevention and cure in resource-constrained shantytowns in Lima, Peru for: the Community Randomized Evaluation of a Socioeconomic Intervention to Prevent TB (CRESIPT) project.

**Methods:**

Newly-diagnosed TB patients from study-site healthposts were eligible to receive the intervention consisting of economic and social support*.* Economic support was provided to patient households through cash transfers on meeting the following conditions: screening for TB in household contacts and MDR TB in patients; adhering to TB treatment and chemoprophylaxis; and engaging with CRESIPT social support (household visits and community meetings).

To evaluate project acceptability, quantitative and qualitative feedback was collected using a mixed-methods approach during formative activities. Formative activities included consultations, focus group discussions and questionnaires conducted with the project team, project participants, civil society and stakeholders.

**Results:**

Over 7 months, 135 randomly-selected patients and their 647 household contacts were recruited from 32 impoverished shantytown communities. Of 1299 potential cash transfers, 964 (74 %) were achieved, 259 (19 %) were not achieved, and 76 (7 %) were yet to be achieved. Of those achieved, 885/964 (92 %) were achieved optimally and 79/964 (8 %) sub-optimally.

Key project successes were identified during 135 formative activities and included: strong multi-sectorial collaboration; generation of new evidence for TB-specific cash transfer; and the project being perceived as patient-centred and empowering.

Challenges included: participant confidence being eroded through cash transfer delays, hidden account-charges and stigma; access to the initial bank-provider being limited; and conditions requiring participation of all TB-affected household members (e.g. community meetings) being hard to achieve.

Refinements were made to improve project acceptability and future impact: the initial bank-provider was changed; conditional and unconditional cash transfers were combined; cash transfer sums were increased to a locally-appropriate, evidence-based amount; and cash transfer size varied according to patient household size to maximally reduce mitigation of TB-related costs and be more responsive to household needs.

**Conclusions:**

A novel TB-specific socioeconomic intervention including conditional cash transfers has been designed, implemented, refined and is ready for impact assessment, including by the CRESIPT project. The lessons learnt during this research will inform policy-makers and decision-makers for future implementation of related interventions.

**Electronic supplementary material:**

The online version of this article (doi:10.1186/s12889-015-2128-0) contains supplementary material, which is available to authorized users.

## Background

Tuberculosis kills 5000 people per day [1], mostly in resource-constrained settings. TB has long been recognised as an illness inextricably linked with social deprivation and marginalisation [2, 3]. Poverty predisposes individuals to TB [4, 5] and hidden costs associated with even free TB treatment can be catastrophic: exacerbating poverty [6], leading to adverse TB treatment outcome, increasing TB transmission and potentially worsening TB control [7]. Nevertheless, the global model for TB prevention, management and research has been principally focused on biomedical rather than socioeconomic approaches [8, 9]. There is a pressing need to expand the traditional TB control paradigm based on case finding and treatment in order to embrace more holistic approaches that encompass the wellbeing of people and households living with TB and communities affected by TB [10–15]. This vision has been formally acknowledged in the World Health Organisation’s (WHO) post-2015 global End TB Strategy [16] which, for the first time in the modern era of TB control, explicitly identifies poverty reduction strategies, including universal health coverage and social protection, as key pillars of the future global response to TB [16, 17].

Social protection consists of policies and programs designed to reduce poverty and vulnerability by improving people’s capacity to manage social and/or economic risks [18], and includes health insurance, food assistance, travel vouchers and cash transfers [19]. Cash transfers generally provide economic support to impoverished people with the aim of moving them out of extreme poverty and vulnerability whilst improving human capital [20–25]. Cash transfers are already used to modulate behaviour in HIV/AIDS [26, 27] and improve maternal health [28]. Mitigating poverty-related TB risk factors of TB-affected households using cash transfers may be a cost-effective investment from a societal perspective [29] because it may support TB treatment, improve TB prevention and cure, and potentially enhance TB control [30]. However, there is little operational evidence to guide implementation or evaluate the impact of TB-related socioeconomic support including cash transfer interventions [15, 19–21, 31–40].

For over a decade, our research group (www.ifhad.org) has worked with TB-affected households in the shantytowns of Callao, Peru. From 2007 to 2011, we conducted an assessment of Innovative Socioeconomic Interventions Against TB (ISIAT) [39]. The interventions had two dimensions: i) education, community mobilization and psychosocial support to increase uptake of TB care; and ii) food transfers, microcredit, microenterprise and vocational training to reduce poverty. This intervention increased preventive chemotherapy in household contacts and HIV testing and TB treatment completion in TB patients [39].

Building on the lessons learnt during the ISIAT project, we designed a larger 6-year research project called CRESIPT: a “Community Randomized Evaluation of a Socioeconomic Intervention to Prevent TB” to test for impact on TB control. This paper aims to describe the challenges of implementation, lessons learnt and refinement of this complex socioeconomic intervention to control TB. The paper focuses on set up of a TB-specific cash transfer scheme, and thus aims to provide research groups, NGOs, civil-society representatives, policy-makers, stake-holders and the wider TB community with an interim guidance document concerning the operational logistics of TB-adapted socioeconomic interventions involving cash transfers in resource-constrained settings.

## Methods

### Intervention objectives

The CRESIPT project aims to evaluate a socioeconomic intervention to support prevention and cure of TB in TB-affected households and, ultimately, improve community TB control. The CRESIPT socioeconomic intervention was developed over 7 months in two contiguous suburbs of Peru’s capital, Lima: Ventanilla, 15 peri-urban shantytown communities in which our research group has been recruiting patients to an on-going cohort study for over a decade; [39] and Callao, an area including 17 impoverished urban communities.

### Intervention planning

The CRESIPT project was informed by our previous research [39], extensive expert consultation [19], and a systematic review [20] of cash transfer interventions published in 2011.

**Fig. 1:**
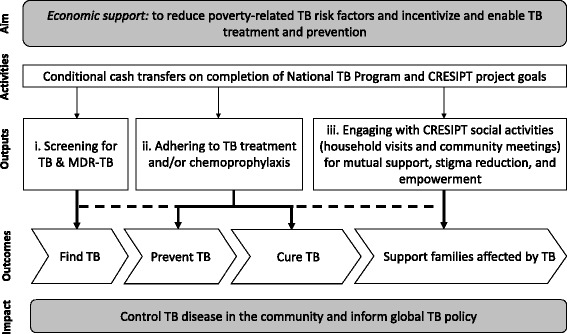
Conceptual framework of the conditional cash transfer scheme within the CRESIPT project

We built upon an *a priori* conceptual framework reflecting the postulated pathways through which the intervention could lead to improved TB control in the study site (Fig. 1). The intervention outputs related to shared CRESIPT project and Peruvian National TB program goals: i. screening for TB in household contacts and MDR-TB in TB patients; ii. adhering to TB treatment and chemoprophylaxis; and iii. engaging with CRESIPT social support activities. Thus, our intervention targeted defined outcomes along the TB causal pathway. In TB patients, we aimed to improve early diagnosis and treatment, provide support to increase adherence to and completion of treatment, and achieve sustained cured. Amongst household contacts living with these TB patients, we aimed to prevent TB.

The previous systematic review of cash transfer interventions was updated in 2014: Medline, Embase, Global Health and HMIC databases were searched from 1st January 2011 onwards using the term **“Tuberculosis/economics” [Mesh] OR “Tuberculosis, pulmonary/economics” [Mesh] OR “Tuberculosis/prevention and control”[Mesh] AND “Economic support” OR “Cash transfers”**. This search found only one randomized controlled evaluation of economic support to improve tuberculosis treatment outcomes [41]. Other necessary and informative literature on economic interventions did not meet inclusion criteria for this systematic review because it either related specifically to HIV/AIDS (such as the IMAGE study) [42] or was limited by having no control group or impact assessment [17, 43].

**Fig. 2:**
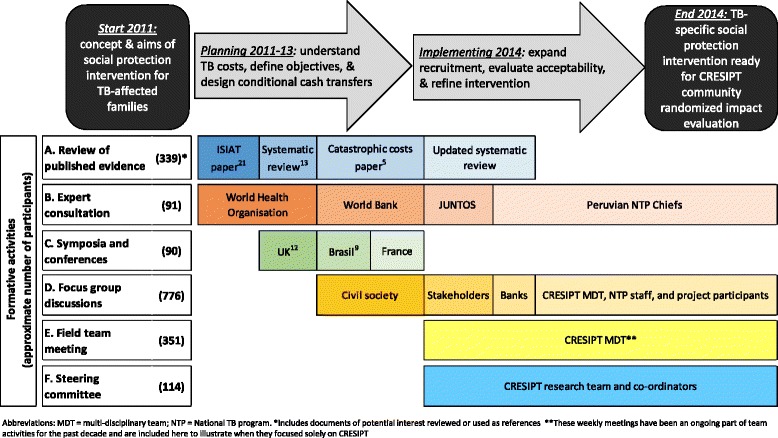
Flow diagram of CRESIPT project activities during planning, implementation and refinement of the social protection intervention

A consultation process was undertaken to inform the project and its scope: a total of 135 formative activities were conducted including multi-sectorial meetings, focus group discussion (FGDs), semi-structured interviews, evidence reviews and other expert consultations (Table 1 and Fig. 2).

**Table 1:** CRESIPT consultation process

Formative activities	Attendees	Number performed	Number of participants^a^	Notes/details
A. Analysis of evidence	CRESIPT project research team and international collaborators from Imperial College London, London School of Hygiene & Tropical Medicine, and John Hopkins School of Public Health	3	28	Analysis and publication of ISIAT project results in 2011 [39] WHO-commissioned systematic review of conditional cash transfer schemes’ impact on TB control in 2011 [20] plus updated review in 2014
B. Expert consultation	Peruvian National TB program chiefs	10	8	Steering meetings with regional and national TB Program coordinators
	JUNTOS (www.juntos.gob.pe) (Peruvian conditional cash transfer program)^b^	1	5	Discussed logistics and minimal impact evaluation of conditional cash transfers for health and education to female heads of rural households [5]
	WHO Stop-TB partnership	3	5	Ongoing meetings and site visits
	World Bank	2	3	Ongoing meetings with senior World Bank economists especially relating to cost-effectiveness considerations
C. Symposia and conferences	International multi-sectoral researchers (including World and Pan-American Health Organisation members)	3	30	“Social protection interventions for TB control”, UK, 2012 [19] WHO led policy consultation on social protection for TB in Brazil, 2013 [16] TB Union World Lung Health conference in France, 2013
D. Focus Group Discussions (FGDs)	CRESIPT multidisciplinary team	9	10	www.ifhad.org FGDs with the CRESIPT field team research personnel
	Ex-TB patient civil society “LUPORFAT”	4	13	Registered “Junta Directiva” (board of directors) of ex-TB patient community representatives “Lucha Por Familias Afectadas Por TBC” www.prisma.org Structured interviews and FGDs
	Key NGO Stakeholders	4	5	
	CRESIPT project participants	19	20	Including participatory community meetings and training of facilitators
	Peruvian National TB program health post staff	18	12	Multi-disciplinary teams: co-ordinators, doctors, nurses and technicians
	Banks	6	5	Account executives and social inclusion department representatives
E. Field team meetings	CRESIPT multidisciplinary team	34	11	Covered operational field logistics and acceptance of the intervention
F. Steering committee	CRESIPT multidisciplinary team & international Collaborators	19	6	Twice monthly committee review of published literature (including systematic review) and discussion of financial, methodological and statistical design issues and potential intervention improvements
TOTAL	NA	135	NA	NA

While JUNTOS may be TB-inclusive (i.e. some TB patients will receive incentives as they are below this poverty threshold), it is neither TB-sensitive nor TB-specific [20]

*FGD* focus group discussion

^a^mean average

^b^We were unable to integrate our urban TB-specific intervention with JUNTOS’ existing rural cash transfer scheme

Table 2 summarises the critical review of the available evidence that occurred during the planning process, and the manner in which this review subsequently informed the main operational design and implementation decisions relating to some of the main aspects of the cash transfer intervention, including: existing cash transfer schemes, conditionality and transfer size.

**Table 2:** Available evidence and CRESIPT project operational decisions relating to cash transfers

	The available evidence - what do we know?	Operational decisions for implementation of the CRESIPT project intervention
Cash transfer schemes	• Cash transfer schemes were implemented in Latin America in the 1990s to tackle the socioeconomic consequences of financial crises [21]. Schemes include: Bolsa Familia (Brasil, 1995); Oportunidades (Mexico, 1997); Red de Protección Social (Nicaragua, 2000–2005); Bono de Desarrollo Humano (BDH Ecuador, 2004); Red Solidaria (El Salvador, 2005); and JUNTOS (Peru, 2005)	• We investigated the use of food or other vouchers/cards but found very few existing systems in the study site. Those that were in place could only be redeemed in supermarkets (felt in FGDs to be inappropriate for the study population due to infrequent use, limited access and higher costs of goods)
	• Our systematic review revealed only one controlled trial of TB-specific cash transfers from South Africa that showed no significant increase in successful TB treatment outcome [41]. During treatment, vouchers (15 US Dollars) that could be exchanged for foodstuffs were given to patients by local health post nursing staff. The authors opted for vouchers over cash due to: posing a lower security risk; not being able to be spent on unhealthy items such as alcohol or cigarettes; being easy to monitor; and “public health sector clinics would not have bank accounts, making electronic transfers difficult” [41]	• Based on our experiences and the limited published evidence, we opted for a bank cash transfer scheme. Bank transfers reduce the likelihood of fraud, robbery or security risk (a concern in impoverished shantytowns in Lima, Peru) [37, 41] and are a reliable way to maintain accurate transfer records for cost-effectiveness analysis. We also felt opening a bank account and having freedom of choice to decide on how transfers were spent was empowering [44]
		• We decided not to impose conditions on how the cash transfers were spent. Successfully funded social protection interventions related to TB (especially MDR TB) have mainly focused on mitigating non-medical direct costs associated with having TB such as food or travel [17, 44]. There is some evidence that even when money rather than food vouchers is given as a form of social protection, it is commonly spent on food and travel costs anyway [45]
Conditionality of cash transfers	• Cash transfers can be unconditional, conditional (requiring specific behavioural, education or health actions) or combined [46, 47]	• Perú has an exemplary, well-established and organised National TB program. Learning from ongoing collaboration with regional heads of the TB program, we decided that our cash transfers conditions would relate to National TB Program treatment and prevention goals and selected project activities
	• Unconditional cash transfer schemes include: Ecuador’s BDH targeting those below poverty threshold or by location; [48, 49] and a village bank loan scheme for TB-affected households in Cambodia [50]	• We chose to use conditional cash transfers that mixed both hard and soft conditions to be more inclusive: “hard” in that if participants met the condition with “perfect behaviour” then a double cash transfer was provided and “soft” in that if participants met the condition with adequate behaviour, then a simple cash transfer incentive was provided (Fig. 4a and b)
	• Conditions can be “hard” (if the condition is not met, the transfer is not made) or “soft” (less stringent conditions where transfers may be made even when a condition is unmet). Soft conditionality may be preferable in settings with poor healthcare infrastructure [21, 46, 51]	
How much cash to give	• Minimal evidence exists on the size of cash transfers. In Latin America, total amounts have varied widely in previous projects: 6-10% of annual income in Ecuador; [49] 21.8% in Mexico; and 29.3% in Nicaragua [47]	• We aimed to establish an amount for the cash transfers that was too small to act as a perverse incentive [34, 35], but large enough so that poverty-related TB risk factors in TB-affected households were reduced and the households were both incentivized and enabled to achieve National TB program and project goals
When to give cash	• Most initiatives deal more with poverty than a finite illness such as TB, so evidence of duration and frequency of TB-specific cash transfers is scarce. Longer duration and more frequent cash transfers may have greater impact in TB-affected households [31]	• We decided to provide the majority of the cash transfer incentives in the intensive treatment phase (the first 2 months of treatment) and to continue monthly cash transfers specific for treatment adherence throughout treatment. This meant the intervention was designed to increase equity for people with TB-HIV co-infection and MDR TB whose treatment lasted longer than 6 months
	• Our previous work in the study setting showed that hidden TB costs were mainly incurred pre-diagnosis or early in treatment [7]	

Thus the planning process involving previous research, extensive expert consultation and systematic reviews of cash transfer interventions led to the design of a novel socioeconomic intervention that aimed to be locally-appropriate, feasible and sustainable and consisted of:

- economic support: conditional cash transfers to reduce TB vulnerability, incentivise and enable care; and
- social support: household visits and participatory community meetings for information, mutual support, stigma reduction and empowerment.

The participatory community meetings, which are reported separately, consisted of an interactive educational workshop concerning issues surrounding TB and household finances, and a “TB Club” in which participants shared TB-related and other experiences in a support group format specifically adapted to the local setting.

### Acceptability

To characterise operational challenges and the participants’ perspectives, we performed an acceptability assessment using a mixed-methods approach. This involved the collection of quantitative and qualitative data from participants, a civil society group of ex-patient community representatives, CRESIPT project staff and local and regional Peruvian TB Program staff and co-ordinators.

### Ethical approval

Approval was granted by the ethics committees of the Callao Ministry of Health, Peru; Asociación Benéfica PRISMA, Peru; and Imperial College London, UK. All interviewed participants gave written informed consent to participate in the study and for subsequent publication of anonymised data.

### Sample size

The main outcome of this preliminary work of the CRESIPT study (reported elsewhere) was completion of TB chemoprophylaxis in household contacts of TB patients. TB patients had an average of five contacts and 25 % of those eligible for TB chemoprophylaxis completed it [39]. Therefore, *a priori*, we calculated that 312 patients would give 80 % statistical power to detect a 33 % increase in the primary outcome comparing intervention versus control households with two-sided 5 % significance. The 312 patients recruited were randomly assigned in a 1:1 ratio to the intervention arm (normal standard of care from the National TB Program plus socioeconomic intervention) and control arm (normal standard of care from the National TB Program).

## Results: designing and implementing the intervention

### Designing the conditional cash transfers

#### Targeting

To provide evidence to assist national TB programs considering implementing TB-related socioeconomic interventions, our intervention exclusively targeted TB-affected households (i.e. was “TB-specific”) rather than targeting all households living below the poverty line. The reasons for this decision were: encouraging results from the TB-specific ISIAT project; [39] the urgent need for impact assessment and operational evidence for TB-specific socioeconomic interventions; the lack of existing TB-specific or TB-sensitive socioeconomic initiatives with which to feasibly collaborate in Peru; and the achievability of focusing on relatively small numbers of TB patients versus much larger, operationally unmanageable numbers of people at risk of TB in the wider community [52]. In addition, it was expected that by working exclusively with TB-affected families we would generate evidence concerning those sections of the community most at risk of TB.

#### Cash delivery strategy

Cash transfers directly into bank accounts were selected as the most locally-appropriate way to deliver economic support because in the impoverished shantytown communities of the study site there were many: local bank agencies; food or material vouchers had poor accessibility and acceptability; direct cash transfers posed a security risk; and transfers using mobile-phone technology potentially overlooked the most vulnerable patients [53] and were prone to handset loss/theft, damage, or faults.

#### Cash transfer size

Deciding on the size and duration of cash transfers was difficult because this has varied considerably in past projects [47, 49]. Learning from similar regional projects [45, 49], our local catastrophic costs findings [7], and ongoing liaison and site visits from key policy-makers from WHO, Pan-American Health Organisation and the World Bank, we aimed to completely mitigate TB-related direct out-of-pocket expenses, which was expected to be equivalent to 10 % of median TB-affected household annual income in the study site [7]. This amount was: empirically believed to be too small to act as a perverse incentive; [34, 35] affordable for a TB program in a low income country (expert opinion suggests that a socioeconomic intervention that adds less than 50 % to the cost of biomedical treatment but reduces TB risk by 30–40 % would be likely to justify policy change and widespread implementation); [54, 55] large enough so that poverty-related TB risk factors in TB-affected households may be reduced; and that incentivized and enabled TB-affected households to achieve the shared goals of the Peruvian National TB Program and CRESIPT project.

#### Cash transfer timing

We designed the intervention so that cash transfers would be provided throughout treatment but weighted towards the first 2 months, when TB-affected households incur the majority of hidden costs (Additional file 1: Figure S1a and b) [7, 56, 57].

#### Cash transfer conditions, levels and responsiveness

**Fig. 3:**
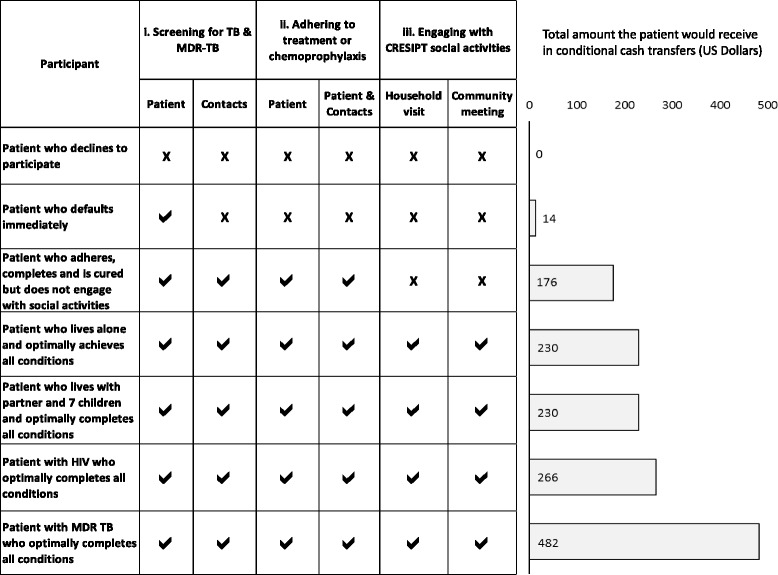
Cash transfer received by participants in seven different potential scenarios during intervention implementation. Note: Typically in Peru, treatment of TB in people with non-MDR TB has a duration of 6 months, in people with HIV-TB co-infection treatment lasts 9 months, and in people with MDR TB treatment lasts 24 months. Key: **✓** = condition optimally achieved and double incentive cash transfer provided; **X** = condition not achieved thus no incentive cash transfer given/paid

We stratified cash transfer incentives into “double” and “simple” incentives. Double incentives were made for meeting the condition “optimally” (i.e. monthly adherence missing less than two daily tablets). Simple incentives were made for meeting a condition “acceptably” (i.e. monthly adherence in which two or more tablets had been missed but the patient had not abandoned treatment). Figure 3 summarizes seven different potential scenarios of TB patients and the total amount of cash transfer incentives they would receive. Were a participant with non-MDR TB to receive all the double incentives available throughout treatment, they would receive a total of 230 US Dollars; for all simple incentives, they would receive a total of 115 US dollars (Additional file 1: Figure S1a and b). In situations in which TB treatment routinely extended beyond 6 months, such as HIV-TB co-infection (9 months) or multi-drug resistant (MDR) TB (18 to 24 months), cash transfers continued throughout the duration of treatment. The decision to stratify simple and double incentives was taken in order to encourage a positive feedback loop of behaviour change through graded incentives whilst increasing the opportunity for vulnerable patient groups to receive cash transfers even when they could not achieve conditions optimally.

### Implementation of the conditional cash transfers

#### Banks

Of 10 banks visited, formal meetings were organised with four that aimed to: create a relationship with the bank to achieve sustainable cash transfers throughout the study; identify charge-free appropriate accounts; create a “virtual” way of opening accounts to minimize paperwork, time spent “in branch” and travel-related patient costs; establish a mutually suitable day on which to accompany patients to open accounts; and to clarify the bank’s accessibility in our study sites (i.e. branches and agencies).

The banks we consulted raised similar concerns about the proposed intervention, including: infection risk; cash transfer flow; and difficulties opening accounts with patients who have no national identification, fixed abode, or are illiterate. We initially chose one bank that appeared to be more likely to overcome these issues because it had a social inclusion department with previous involvement in successful microfinance initiatives.

#### Opening bank accounts

Recruited patients with a negative sputum smear microscopy test (indicating low infectiousness) were accompanied by our project staff to open a bank account. The account holder’s details were then relayed to our project office with a copy of the bank’s original documents. In the case that the patient was a minor, did not have legal capacity, wished for another household member to be the named bank account holder, or had prolonged sputum smear positivity, then a household member was selected by the patient or household to be the named bank account holder. Patient transport and time costs were reimbursed by our project.

#### Cash transfer administration

The patient’s incentive card (Additional file 1: Figure S1a and b) was updated by the field nurses when each condition was achieved. Confirmation of completion was made through liaison with the patient, review of CRESIPT project records (e.g. participatory community meeting attendance) and Peruvian National TB Program records and treatment cards (e.g. medication adherence verification). Signed incentives cards were returned to a project administrator who double digitized the data. Thus, a weekly list of patients, their bank account details and required transfers was generated. The same day, this list was submitted electronically to a member of the social inclusion department of the bank, and the virtual transfers made. The transaction codes and receipts generated were double digitized in the CRESIPT project database and delivered to the patients in the health post by the CRESIPT field nurses.

### Recruitment

From February to August 2014, we expanded project activities from 2 to 32 communities. As per the *a priori* sample size calculations and study protocol, 312 consecutive TB patients from the study site were invited to participate of whom 149 were randomized to receive the socioeconomic intervention. 12/149 (8 %) patients declined to participate, 2/149 (1 %) died prior to recruitment, and 2/149 (1 %) were recruited and subsequently did not complete follow up. Thus, 133/149 (89 %) were recruited and participated throughout the study period. The number of patients declining to participate was higher in urban than in peri-urban communities (15 % [95 % CI 6–23] versus 5 % [95 % CI 1–10 %] respectively, *p* = 0.04). Of the 133 participants, 9/133 (7 %) had MDR TB, 5/133 (4 %) were HIV positive, and 7/133 (5 %) were diabetic.

#### Cash transfers achieved up to 1st July 2015

**Fig. 4:**
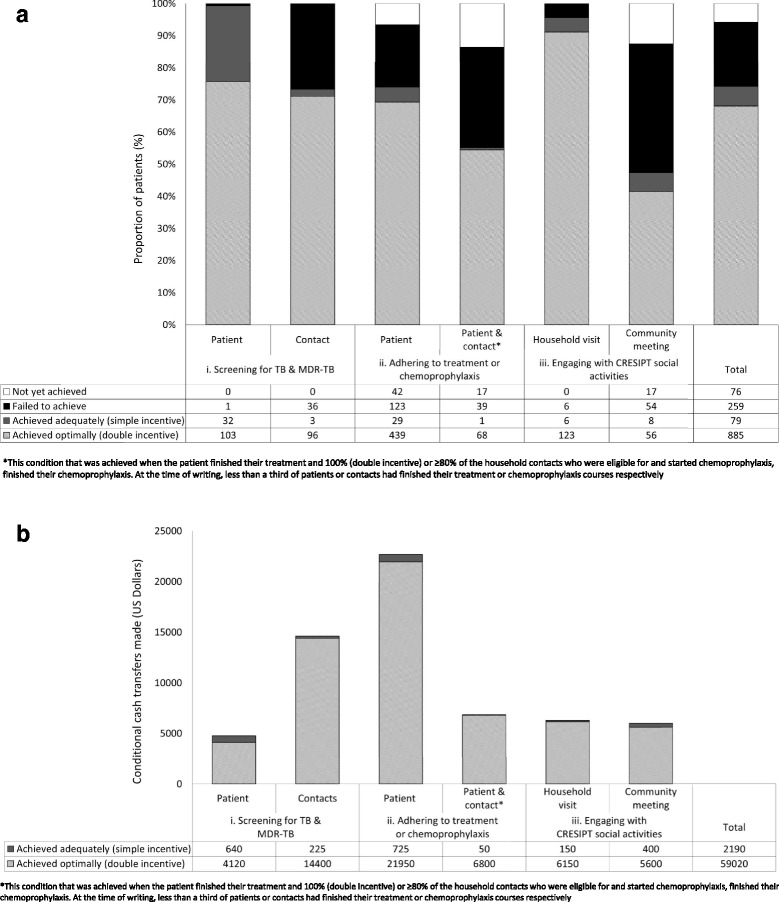
**a** Proportion of patients optimally achieving (double incentive), adequately achieving (simple incentive) and not yet achieving project conditions. **b** Total amount provided to patients by conditional cash transfers in total and for each condition achieved

Of 1299 potential cash transfers, 964 (74 %) were achieved (of these, 885/964 [92 %] were achieved optimally and 79/964 [8 %] sub-optimally), 259 (19 %) were not achieved, and 76 (7 %) were yet to be achieved. Thus, 964 conditional cash transfers totalling 61,120 Peruvian Soles (20,373 US dollars) were made to TB-affected households for meeting the conditional goals of the Peruvian National TB Program and CRESIPT project (Fig. 4a and b). The average cash transfer amount received by each TB-affected household over the course of the intervention was $173 USD.

## Discussion: lessons learnt and persisting knowledge gaps

The implementation and acceptability assessment identified strengths and limitations of our theoretical approach during the design of CRESIPT. The lessons that emerged are grouped into successes, challenges and refinements in Table 3, which aim to inform the design of future studies and, ultimately, allowed us to identify persisting knowledge gaps in this field (Table 4).

**Table 3:** Successes, challenges and refinements of the cash transfer incentive dimension of the socioeconomic intervention

	Successes	Challenges	Refinements
New evidence	New experience and evidence was generated that TB-specific cash transfers for TB-patients were feasible in this study setting	There was a lack of available evidence and thus clarity when prioritising the output of the cash transfers in these TB-affected households. Thus, deciding on the cash transfer amounts and timing was difficult	Following previous and updated analysis of hidden costs and income of TB-affected households [7], cash transfer amounts were increased with the aim of reducing their poverty-related TB risk factors
Collaboration	There was strong multi-sectorial collaboration with Peruvian National TB Program and bank staff, allowing multiple, virtual cash transfers to be made and recorded, reducing fraud and security risks	Account maintenance charges were introduced by the bank during implementation of the intervention and delays in cash transfers eroded participants’ trust in the project	We changed our bank service provider: the new bank had better accessibility and no charges. We self-imposed penalties on our project for late cash transfers (participants gained additional transfers)
Cash transfers	Cash transfers lasted throughout treatment, increasing equity for people with TB and HIV co-infection or MDR TB, whose treatment duration extended beyond 6 months	As a research team, we had limited experience of cash transfer interventions or working with new urban study communities	Achieving a balance between operational simplicity and complex TB-affected household needs was challenging
	Opening a bank account was a first-time experience for many of the participants and qualitative participant feedback suggested that this was perceived as an empowering action, especially for female members of the household who have previously been shown to be a vulnerable subpopulation in the study setting [5]	Feedback suggested that patients would prefer immediate gratification for completion of conditions rather than delayed cash transfer bank payments	Immediate incentives were provided for attending participatory community meetings (including food baskets and high-quality vouchers documenting the date and amount owed to the participant)
		Project conditions requiring all members of the TB-affected household to participate were poorly achieved and not equitable due to different household sizes	We combined conditional and unconditional cash transfers. Conditions requiring household participation were altered to be responsive to household size: incentives given were refined to be given per household member involved
Inclusiveness and high risk groups	The intervention was holistic and household-centred because, in addition to cash transfers, it provided community meetings consisting of educational workshops (covering themes such as TB treatment, transmission, prevention and also financial themes such as responsible household budgeting in an interactive manner) and TB Clubs (mutual support aiming to reduce stigma and increase empowerment, reported separately)	“High risk” patients in more urban communities were difficult to engage with (especially the formerly-incarcerated, drug- or alcohol-dependent and gang members)	Participatory community meetings for patients with MDR TB were established and increasing social support was provided to other high risk patients (including the homeless, drug or alcohol dependent, those with poor adherence and/or lack of engagement with our project)

**Table 4:** Lessons learnt and persisting research gaps

Lessons learnt	Research gaps
Social protection interventions for TB control require inter-sectorial collaborations	• What are the most effective and cost-effective partnership models for welfare and TB control bodies?
	• What are the best ways to integrate poverty reduction strategies and biomedical activities for TB control?
TB-specific conditional cash transfers are feasible and safe, but logistically complex	• What is the role of conditions in achieving the intervention objectives?
	• Are conditional, unconditional or combined cash transfers preferable and how does this depend on the settings in which the cash transfer program is implemented? [62]
	• What conditions are too hard to achieve for TB patients despite being well rewarded?
	• What is the best way to balance the conditions for the cash transfers in order that they reflect both the priorities of patients and their households, and the priorities of researchers and policy makers? [63]
	• What is the role of the size and timing of cash transfers on the impact of the intervention?
	• What is the effectiveness and cost-effectiveness of different delivery mechanisms?
TB-specific conditional cash transfers can be challenging to deliver to difficult-to-reach populations	• What are the optimal ways to adapt conditional cash transfer settings targeted at hard-to-reach populations in challenging urban environments characterised by violence, drug-addiction and marginalisation?
	• Should social protection interventions only be offered to high-risk patients or is it more cost-efficient to offer them to all patients plus an enhanced intervention to high-risk patients?
	• Is cash without social support sufficient to reach high-risk-patients or is social support necessary?
Health and financial management education are necessary and ethically appropriate	• Would cash transfers have the same impact even without an educational component?
	• What is the empowering factor of the cash transfers to TB patients: 1) receiving cash; or 2) being acknowledged and seen as individuals with rights and needs?
	• What is the aspect of the social protection intervention most likely to impact on TB prevention and cure: a) the economic dimension of cash transfers; b) the social dimension of home visits and community meetings; or c) both?

### Successes

This project generated evidence that conditional cash transfers to TB patients were logistically achievable in impoverished shantytown communities of Lima, Peru. The intervention considerably supplemented small monthly food baskets given unconditionally to TB patients by the Peruvian National TB Program.

Through regular steering meetings, focus group discussions and contact in the health posts, strong collaboration was achieved between our team, banks and the Peruvian National TB Program:

*“The CRESIPT project and Peruvian National TB Program are complementary and should continue to support each other in a common goal.”* [Peruvian National TB Program Regional Chief].

Such ongoing collaboration and adaptation to stakeholder and participant feedback helped the project to be more locally-appropriate, responsive and patient-centred.

The conditional cash transfers were facilitated by multi-sectorial collaboration including with the bank’s social inclusion department. Multiple, regular, simultaneous, virtual cash transfers were achieved through online banking that generated a digital record, reducing the likelihood of fraud. Because field team staff were not directly carrying or giving cash or cash-equivalents (such as cash vouchers or cheques), cash transfers were a secure method of providing incentives. The majority of participants did not have bank accounts [58] and some patients described the act of opening a bank account as empowering:

*“…especially for female patients, who are not normally the financial decision-makers of the households in these communities”* [CRESIPT Project Nurse Technician].

The socioeconomic intervention was holistic and household-centred with the economic dimension of cash transfers being complemented by social support activities including household visits and participatory community meetings [59]. In addition to TB prevention and control messages, an educational component was an important element of the participatory community meetings, in which participants were involved in educational activities concerning: managing a household budget; spending and saving responsibly; and meeting the conditions for cash transfers. This TB-related and financial education was highly rated by participants and perceived as an ethically sound accompaniment to cash transfers by CRESIPT project staff and TB-affected households:

“*I understood and learnt more, and saw that I was not alone”* [TB-Affected Household Member];

“*The meetings generated good solidarity and camaraderie*” [TB-Affected Household Member].

Our experience is consistent with reports that financial incentives should be complemented by education or “social marketing” if health objectives are to be achieved. Further research is needed to investigate the relative importance of health and financial education on the impact of cash transfer interventions aiming to improve health.

### Challenges and refinements

The lack of published evidence of similar studies was particularly challenging for the implementation of TB-specific cash transfer incentives in resource-constrained communities.

#### Cash transfer targeting

The significantly higher number of patients declining to participate seen in the urban rather than peri-urban communities may have been due to the fact that CRESIPT project activities were new in this area or reflect distinctions between these communities:

*“We don’t fully understand the demographic differences and challenges between the urban and peri-urban communities”* [CRESIPT Project Investigator]

The field staff reported that some patients were not willing to participate because: i) they thought that CRESIPT project staff were part of a governmental body; ii) they did not want to register their true address with either the TB program, a bank, or the CRESIPT project in order to keep *“under the radar”* [CRESIPT Project Nurse] especially those formerly-incarcerated or involved with “*pandillas*” (drugs gangs); iii) they did not wish our team to visit their home because their household was unaware of their diagnosis or they frequently moved location; or iv) the incentives were insufficient to match the money they lost for participating in project activities and, more importantly, continuing on their treatment. In addition, patients with recognised “high-risk” factors for treatment default such as prolonged treatment courses (e.g. MDR TB and/or HIV), mental illness, illegal drug use, homelessness, or being formerly-incarcerated were difficult to recruit and retain. Because these patients did not consent to participate we could not formally characterise their reasons for declining. This lack of engagement and formal feedback is concerning given that such groups may have benefited most from the intervention.

*“Conditions are appropriate but you need to provide additional support to those people who find it harder to meet those conditions”* [Ex-TB Patient Civil Society Representative]

*“Some patients would never open a bank account because they don’t want to register their name”* [CRESIPT Project Nurse]

*“[A negative aspect of the CRESIPT project intervention is] giving an economic incentive to a patient with drug-dependency and for that matter any other benefit/incentive such as food baskets (which some of these patients sell to buy drugs)”* [Peruvian National TB Program Nurse]

To combat some of these issues, extra care was taken during the informed consent process to reassure potential participants that the CRESIPT project team is a non-governmental research organisation with no connections to the justice system and that no project activity, especially household visits, was mandatory. In an attempt to address the needs of participants with HIV and/or MDR TB, we explicitly specified that cash transfers for adherence were provided throughout treatment, regardless of treatment duration. This longevity meant that TB-affected household support, staff-household relationship and financial benefits of the cash transfers were refined to be more equitable and responsive to the ongoing needs of patients with HIV and MDR TB.

Hard-to-reach populations and/or difficult urban settings such as those in which our intervention was implemented, may be characterised by violence, illegal drug use and severe marginalisation that are also associated with TB. These populations and those with comorbidities (such as diabetes, HIV, or mental illness) and/or MDR TB may require differential levels of intervention including prolonged or enhanced conditional cash transfers and social support. Future studies may investigate the barriers, feasibility and impact of delivering TB-specific socioeconomic interventions to challenging, vulnerable groups in such settings.

#### Cash transfer delivery strategy

We changed bank-provider because the initial bank-provider: had limited geographical accessibility; provided inconsistent information “in branch” (resulting in some patients opening accounts with maintenance fees); was reported during feedback sessions to have been stigmatizing towards patients, not due to TB (the branch staff were unaware of patients’ diagnoses) but possibly due to other sociocultural factors such as poverty or appearance; and introduced account maintenance charges to previously charge-free accounts.

*“Some patients lost faith in the project due to the hidden bank charges”* [CRESIPT Project Nurse].

The new bank-provider, while not having a specific social inclusion department, provided improved coverage and accessibility because of a greater density of agency micro-branches in local shops that facilitated participant transactions. While the new bank-provider overcame the challenges described above, these experiences have led us to question whether banks are the most appropriate delivery strategy. Indeed, conditional cash transfer programs in Sub-Saharan Africa have predominantly used specified pay points to pay participants in cash rather than banks which may be less accessible to the poor and may have user fees [60]. However, banks have been the favoured partner agent in existing conditional cash transfer programs in Peru (JUNTOS), Brasil (Bolsa Familia) and Mexico (Progresa) with the co-ordination of national cash transfer programs being centralised through national banks in these countries [60]. This level of coordination may only be suitable in countries with comparatively developed financial infrastructure, information and communications technologies, and accessibility to bank branches or micro-agencies. We have reviewed other modalities of conditional cash transfers in greater detail elsewhere [20]. Future research into implementation of socioeconomic interventions may compare the effectiveness and cost-effectiveness of cash delivery mechanisms including mobile phone vouchers, mobile banking, automated or other pay points, or innovative strategies, for which rigorous evidence is currently lacking. To achieve optimal impact, implementers of conditional cash transfer programs may work more closely with the local communities and civil societies to establish how a program can be adapted to be appropriate and acceptable in that specific setting.

#### Cash transfer size

During focus group discussions, the internal research committee debated what the most important objective of cash transfers is: mitigating TB-related costs; avoiding catastrophic costs; or reducing poverty-related TB risk factors. This confounded deciding the cash transfer amounts. To address this challenge, we analysed TB patients’ costs, which demonstrated that direct out-of-pocket expenditure was 10 % of that household’s annual income [7]. These results, together with additional data characterising annual household income for TB-affected households in the study site, informed the cash transfer amount necessary to match direct out-of-pocket expenditure and subsequently avoid catastrophic costs. The optimal cash transfer size is likely to depend on the intervention setting and proposed outcomes of the intervention. This will require baseline evaluation of local TB-related costs prior to planning for and implementing cash transfers of suitable amounts. Further research is required to evaluate how cash transfer size affects intervention impact and cost-effectiveness [61]. It is noteworthy that the strategy we adopted in this research was a “costs-mitigation” rather than a “poverty-reduction” strategy and involved a cash transfer amount that were appropriate and feasible for the local setting. While long-term poverty reduction would be an appealing additional goal, this would likely require greater socioeconomic support than national TB programs could afford.

#### Cash transfer timing

During focus group discussions, the delay between incentivized behaviour and cash transfers was noted as a barrier to achieving project conditions due to the lack of a tangible “reward” and accompanying positive reinforcement loop. For example, when a household attended a participatory community meeting, it could be 1 to 2 weeks before they received the corresponding cash transfer.

*“Patients want immediate and tangible gratification on the same day as they complete their condition”* [Ex-TB Patient Civil Society Representative].

Cash transfers were initially delayed due to the flow of information from the field, to the research office, to the bank. While cash transfer flow improved during the project, patients and households stated that such delays made household budgeting difficult:

*“Due to the initial cash transfer delays, some patients didn’t get the money when they most needed it”* [CRESIPT Project Nurse].

Consequently, we increased the speed of the cash transfers and plan to instigate a system during CRESIPT in which on the *same* day that a household attends a community meeting, they receive a small high-protein food basket and a high-quality certificate-like voucher detailing the amount and date by which the cash transfer would be made.

Participants reported that they would prefer to receive cash transfers at the beginning of the month for their adherence in the subsequent month rather than wait until the end of the month. As has been reported in other settings [45], the waiting was perceived as financially stressful and, on occasion, demoralising. Learning from this setting-specific qualitative feedback, in the planned CRESIPT study in these same communities, we will combine unconditional monthly cash transfer provided to all TB patients taking treatment with supplementary conditional cash transfers for meeting CRESIPT project and National TB Program conditions. Furthermore, we have self-imposed penalties on our project if incentives do not reach the patient’s bank account within 1 week of confirmation that the condition has been met. Specifically, if a delay occurs, their cash transfer is doubled.

#### Cash transfer conditions, levels and responsiveness

Project conditions requiring involvement of “100 %” of the TB-affected household in order to receive the cash transfer (e.g. attendance at participatory community meetings) were hard to achieve. In addition, the amount of these cash transfers was fixed and independent of household size (see Fig. 3) and thus felt to be inequitable because larger households received a lower cash transfer amount per household member. There was, therefore, a perceived challenge in balancing operational simplicity (e.g. fixed-amount incentives) while responding to patient household needs (e.g. variable incentive depending on household size). Consequently, on the basis of this qualitative evaluation of the implementation process, we suggest designing relevant incentives to be more equitable and responsive to household size: a fixed amount added to the patient’s cash transfer for *each member* of their household on completion of the condition. In this way, larger households will receive the same amount per household member as smaller households.

## Conclusions

A novel TB-specific socioeconomic intervention was: designed through multi-sectoral collaboration coupled with evidence from a systematic review; refined to meet patient and household needs during implementation through community participation, engagement and acceptability feedback; and proved to be feasible in an impoverished, challenging environment. The intervention is now ready for impact assessment, including by the CRESIPT project. Further lessons from CRESIPT will aim to assist TB control programs to effectively implement the recent global policy change of including socioeconomic support as part of TB control activities.

## Additional file

Additional file 1: Figure S1a. Details of the operational conditions to meet in order to receive double incentives. b: Details of the operational conditions to meet in order to receive simple incentives. (ZIP 492 kb)
