# Supplementary material for: Designing and implementing a socioeconomic intervention to enhance TB control: operational evidence from the CRESIPT project in Peru
Source: BMC Public Health. 2015 Aug 21;15:810. doi: 10.1186/s12889-015-2128-0 (PMC4546087; doi:10.1186/s12889-015-2128-0)
Supplement: Additional file 1: Figure S1a. — Details of the operational conditions to meet in order to receive double incentives. b: Details of the operational conditions to meet in order to receive simple incentives. (ZIP 492 kb) [file 12889_2015_2128_MOESM1_ESM.zip › 7052233431544554_add1.pdf]

		DOUBLE INCENTIVES	
Output	Condition	What the person with TB and their household members are required to do to receive a DOUBLE incentive	What each TB-affected family will receive in Soles (Dollars)
i. SCREENING FOR TB AND MDR-TB	Patient: sputum sample	Give a sputum sample prior to starting treatment and complete the questionnaire with the research nurse. The national TB program must have registered the person with TB in their health post's TB patient register	40 (14)
	Contact: TB screening	All (100%) of the people registered as living in the same house as the person with TB attend their medical appointment or give a sputum sample (that has a result) to rule out TB. Those people who need to take medicine to prevent TB (chemoprophylaxis) or TB treatment, must have started it. Contacts who do not need to take such treatment must be confirmed in the medical treatment cards	150 (54)
ii. ADHERENCE TO TB TREATMENT OR CHEMOPROPHYLAXIS	Patient: adherence to treatment	SENSITIVE/NON-MDR PATIENTS (INCLUDING THOSE WITH HIV), DURING THE FIRST 50 DOSES OF TREATMENT: each 25 doses (approximately each month) of TB treatment taken missing no more than 2 doses	50 (18)
		MDR PATIENTS, DURING THE FIRST 150 DOSES OF TREATMENT (APPROXIMATELY 6 MONTHS): each 25 doses (approximately each month) of TB treatment taken missing no more than 2 doses	50 (18)
		SENSITIVE/NON-MDR PATIENTS (INCLUDING THOSE WITH HIV), AFTER THE FIRST 50 DOSES OF TREATMENT TO THE END OF TREATMENT: each 24 doses (approximately two months) of TB treatment taken missing no more than 1 dose	50 (18)
		MDR PATIENTS, AFTER THE FIRST 150 DOSES OF TREATMENT UNTIL THE END OF TREATMENT: each 50 doses (approximately two month) of TB treatment taken missing no more than 2 doses	50 (18)
	Patient and contacts: completion of TB treatment and chemoprophylaxis	The person with TB completes their TB treatment and all (100%) of the people who live with them who started chemoprophylaxis to prevent TB, finish their chemoprophylaxis	100 (36)
iii. ENGAGE WITH CRESIPT SOCIAL ACTIVITIES	Patient and contacts: home visit	In the first week following recruitment to our project, allow the CRESIPT team to visit the person with TB's house, complete the questionnaire (if necessary), and list all the people who live in the same house as them	50 (18)
	Patient and contacts: community meetings	By 3 months following recruitment, the person with TB and all (100%) of the people that the CRESIPT team listed as living in the same house as them attend at least 1 CRESIPT community meeting	100 (36)
COMPLETE ALL CONDITIONS		The person with TB and all of their contacts complete all the above project conditions including adhering to treatment for the duration of treatment	640 (230)
