# Supplementary material for: Designing and implementing a socioeconomic intervention to enhance TB control: operational evidence from the CRESIPT project in Peru
Source: BMC Public Health. 2015 Aug 21;15:810. doi: 10.1186/s12889-015-2128-0 (PMC4546087; doi:10.1186/s12889-015-2128-0)
Supplement: Additional file 1: Figure S1a. — Details of the operational conditions to meet in order to receive double incentives. b: Details of the operational conditions to meet in order to receive simple incentives. (ZIP 492 kb) [file 12889_2015_2128_MOESM1_ESM.zip › 7052233431544554_add2.pdf]

SIMPLE INCENTIVES

Output	Condition	What the person with TB and their household members are required to do to receive a SIMPLE incentive	What each TB-affected family will receive in Soles (Dollars)
i. SCREENING FOR TB AND MDR-TB	Patient: sputum sample	Give a sputum sample as soon as possible after starting treatment and complete the questionnaire with the research nurse. The national TB program must have registered the person with TB in their health posts TB patient register	20 (7)
	Contact: TB screening	>80% of the people registered to live in the same house as the person with TB attend their medical appointment or give a sputum sample (which has a result) to rule out TB. Those people who need to take medicine to prevent TB (chemoprophylaxis) or TB treatment, must have started it. Contacts who do not need to take such treatment must be confirmed in the medical treatment cards	75 (27)
ii. ADHERENCE TO TB TREATMENT OR CHEMOPROPHYLAXIS	Patient: adherence to treatment	SENSITIVE/NON-MDR PATIENTS (INCLUDING PEOPLE WITH HIV), DURING THE FIRST 50 DOSES OF TREATMENT: each 25 doses (approximately each month) of TB treatment taken missing equal to or more than 3 doses	25 (9)
		MDR PATIENTS, DURING THE FIRST 150 DOSES OF TREATMENT (APPROXIMATELY 6 MONTHS): each 25 doses (approximately each month) of TB treatment taken missing equal to or more than 3 doses	25 (9)
		SENSITIVE/NON-MDR PATIENTS (INCLUDING PEOPLE WITH HIV), AFTER THE FIRST 50 DOSES OF TREATMENT TO END OF TREATMENT: each 24 doses (approximately two months) of TB treatment taken missing equal to or more than 2 doses	25 (9)
		MDR PATIENTS, AFTER THE FIRST 150 DOSES OF TREATMENT UNTIL THE END OF TREATMENT: each 50 doses (approximately two month) of TB treatment taken missing equal to or more than 3 doses	25 (9)
	Patient and contacts: completion of TB treatment and chemoprophylaxis	The person with TB completes their TB treatment and 80% or more of the people who live with them who started chemoprophylaxis to prevent TB, finish their chemoprophylaxis	50 (18)
iii. ENGAGE WITH CRESIPT SOCIAL ACTIVITIES	Patient and contacts: home visit	In the first month following recruitment to our project, allow the CRESIPT team to visit the person with TB's house, complete the questionnaire (if necessary), and list all the people who live in the same house as them	25 (9)
	Patient and contacts: community meetings	By 5 months following recruitment, the person with TB and all (100%) of the people that the CRESIPT team listed as living in the same house as them attend at least 1 CRESIPT community meeting	50 (18)
COMPLETE ALL CONDITIONS		The person with TB and most of the people who live with them complete all the above project conditions including adhering to treatment for the duration of treatment	320 (115)
